# Kell‐Score—A web resource for estimating the immunogenicity of novel Kell blood group variants

**DOI:** 10.1111/bjh.70525

**Published:** 2026-05-14

**Authors:** Gabriele Mayr, Marcus Schmöhl, Maike Bublitz, Christoph Gassner, Andre Franke

**Affiliations:** ^1^ Institute of Clinical Molecular Biology, Christian‐Albrechts‐University of Kiel & University Hospital Schleswig‐Holstein (UKSH) Kiel Germany; ^2^ Institute of Translational Medicine, Faculty of Medical Sciences, Private University in the Principality of Liechtenstein (UFL) Triesen Liechtenstein

**Keywords:** destabilization and antigenicity predictions, Kell blood group system, protein missense variants, webserver

To the Editor,

High‐throughput technologies accelerate the discovery of new blood group variants^1^ and aim at preventing future clinical complications. Thus, tools for predicting the blood group phenotype from genetic data would help haematologists to interpret the significance of new variants for clinical practice, and furthermore, to identify rare blood group carriers. To date, the Kell blood group system includes 38 antigens as approved by the International Society of Blood Transfusion (ISBT) Red Cell Immunogenetics and Blood Group Terminology Working Party.^2^, ^3^

Recently, we analysed the molecular properties of all currently registered *KEL* alleles that encode full‐length variants of the Kell protein as either unexpressed/null (*K*_0_) or diminished/mod phenotype (*K*_mod_) or expressing different antigens.^4^ The insights gained in this study were used to create a catalogue of objective criteria to estimate the potential of a new Kell variant to (i) destabilize the protein, for example, to prevent expression on the erythrocyte surface by hampering stable protein folding, or alternatively, to (ii) create a new antigen on the protein surface that is specifically recognized by immune receptors. With this, a two‐score heuristic measure that combines features typical for a specific phenotype was computed for all theoretically possible Kell missense mutations. These measures are not intended to quantify the immunogenicity of Kell variants,^5^ but rather to create an initial evaluation of the potential molecular effects of newly discovered variants.

Here we provide the results of these predictions to the scientific community through a user‐friendly online resource that includes an intuitive analysis pipeline to explore and evaluate any number of previously uncharacterized Kell missense variants. Amino acid positions of interest can be simultaneously localized in the 3D protein structural viewer. The web interface is accessible at https://kellscore.ikmb.uni‐kiel.de.

For collecting Kell variant characteristics, we used the same dataset of Kell blood group variant characteristics as published in our previous study.^4^ Numerical values applied here for predicting phenotypes are provided in the Supporting Information Data Sheet (Table S1). In brief, a set of 3D protein structural, evolutionary and biophysical parameters was compiled as follows:

- We have observed that the structural location can serve as a first indicator for predicting a phenotype. However, following the molecular analysis of the dataset control variants (denoted as ‘CtrlV’), that are expressed/non‐destabilizing and non‐antigenic Kell variants, we also concluded that additional factors are usually contributing to the phenotype.^4^ As a basic principle, destabilizing protein variants are usually buried, whereas antigenic variants tend to be accessible on the protein surface. Half‐buried and consequently half‐accessible amino acids can contribute to both phenotypes. Further, co‐localization of destabilizing *K*_0_ and *K*_mod_ protein variants indicate structural regions less tolerant to missense mutations, as opposed to the area around the active site and a loosely packed central cavity. Further, a co‐localization of surface antigens may hint to a discontinuous B‐cell epitope. To define structural neighbours, an atomic distance cut‐off of 7 Å between two amino acids was applied.
- Amino acid positions with a protein stabilizing function are often invariant among orthologues (homologues by speciation) and the more remote paralogues (homologues by gene duplication). Forty orthologue sequences and three paralogues from the M13 family of metallopeptidases were aligned to assess sequential and structural conservation.^4^
- Amino acid substitutions are less likely to have an influence on protein fold stability and expression if the properties of wild‐type and variant amino acid side chains are similar. According to our previous analyses,^4^ hydrophobicity and side chain volume, in combination with their burial status, were best suited to distinguish variants among the different Kell classes. In order to assess the similarity between amino acids, we derived numerical values for amino acid properties from the AAIndex Database.^6^
- Some amino acids are over‐represented in ISBT phenotypes: arginine (R), glutamine (Q) and tryptophan (W) in the dataset of Kell antigenic variant and wild‐type residues, and glycine (G) and proline (P) in destabilizing *K*_0_ and *K*_mod_ variants.^4^
- In addition, automated predictions for variant pathogenicity and destabilization were included as previously described (4): MPC, DEOGEN2 and CADD provide pathogenicity prediction scores, and RaSP and AlphaMissense estimate protein structural destabilization.^4^

Based on these Kell variant parameters, we set out to define a weighting scheme for an automated classification/categorization of any protein missense variant. To this end, we designed a two‐score heuristic measure by collecting protein amino acid positional and variant features. Simply, any property that is considered typical for a phenotype is adding up to the final score for predicting Kell (i) destabilization or (ii) antigenicity. Therefore, the two prediction scores of an individual variant are each the sum of added characteristic points. Some features are given more weight than others and can be interdepending (Table 1); further details on scoring conditions are given in the Supporting Information Data Sheet (Table S2). A maximum score of five indicates a strong prediction for the given phenotype, and zero denotes a predicted ‘neutral’ variant of no effect. On the webpage, the two scores are displayed graphically as a five‐point system for a quick visual estimation of the variant.

**TABLE 1:** Selected amino acid site and molecular properties for predicting a mutational effect. [Colour table can be viewed at wileyonlinelibrary.com]

Category	Condition	Destabilizing	Antigenic
Sequential location	At known *K*_0_/*K*_mod_ residue position	2	
	At known ISBT antigen residue position		2
	At position of a CtrlV	−1	−1
	At Thr193 position (site of KEL1)		4
Structural distances (<7 Å)	Near *K*_0_/*K*_mod_ residue position	1	
	Near ISBT antigen residue position		1
	Near Thr193 position (site of KEL1)		2
Location/accessibility	Buried	1	
	Half‐buried	1	1
	Accessible (>12 Å from membrane)		1
	Inaccessible (<12 Å from membrane)		max 1*
	In buried cavity or active site	max 2*	
	At predicted epitope		1
Conservation	Structural (M13 protein family paralogues)	**2**	**1**
	Sequential (≥95% orthologues)	**1**	
Disulphide bonds	Stabilizing Cys‐bridges	3	
Physico‐chemical property	Changes in residue hydrophobicity	**1**	**1**
	Changes in residue side chain volume	**1**	
Amino acid arginine (R)	Often antigenic and destabilizing	**1**	**2**
Amino acid glutamine (Q)	Often antigenic variant position		**1**
Amino acid tryptophan (W)	Very large side chain	**1**	**1**
Amino acid glycine (G)	Very flexible backbone, small side chain	**1**	
Amino acid proline (P)	Very rigid backbone	**1**	**1**
Automated predictions	High MPC pathogenicity prediction score	1	
	RaSP and AlphaMissense destabilization	1	

*Note*: Calculation of heuristic scores: Sum of points (up to five, except *default maximum scores). The two heuristic scores for predicting destabilization and antigenicity are calculated by summarizing individual conditional sub‐scores for respective amino acid site properties and variant features to a maximum of five. Signs in red denote sub‐scores that are only counted when occurring in conjunction with a buried position, in blue with an exposed position. Known ISBT variants get maximum scores for their phenotype. More details on score calculation are provided in the Supporting Information Data Sheet (Table S2).

Abbreviation: ISBT, International Society of Blood Transfusion.

In the web interface, the user can browse along the protein sequence (UniProt accession: P23246, NCBI reference sequence: NP_000411.1) and simultaneously highlight the 3D position of the selected amino acid in the structural model^4^, ^7^ (Figure 1A,B), where it is visualized as ball‐and‐sticks together with residues within a 7 Å radius. These structural neighbours are also highlighted in the sequence. Known Kell phenotypes are colour‐coded as previously applied.^4^ Destabilization and antigenicity scores can be obtained from a pop‐up window by clicking a residue and choosing the variant amino acid of interest, by uploading variants from a local text file or by entering variants in the text box above. In addition, single missense variants that define an ISBT allele can be chosen from a dropdown button.

**FIGURE 1:**
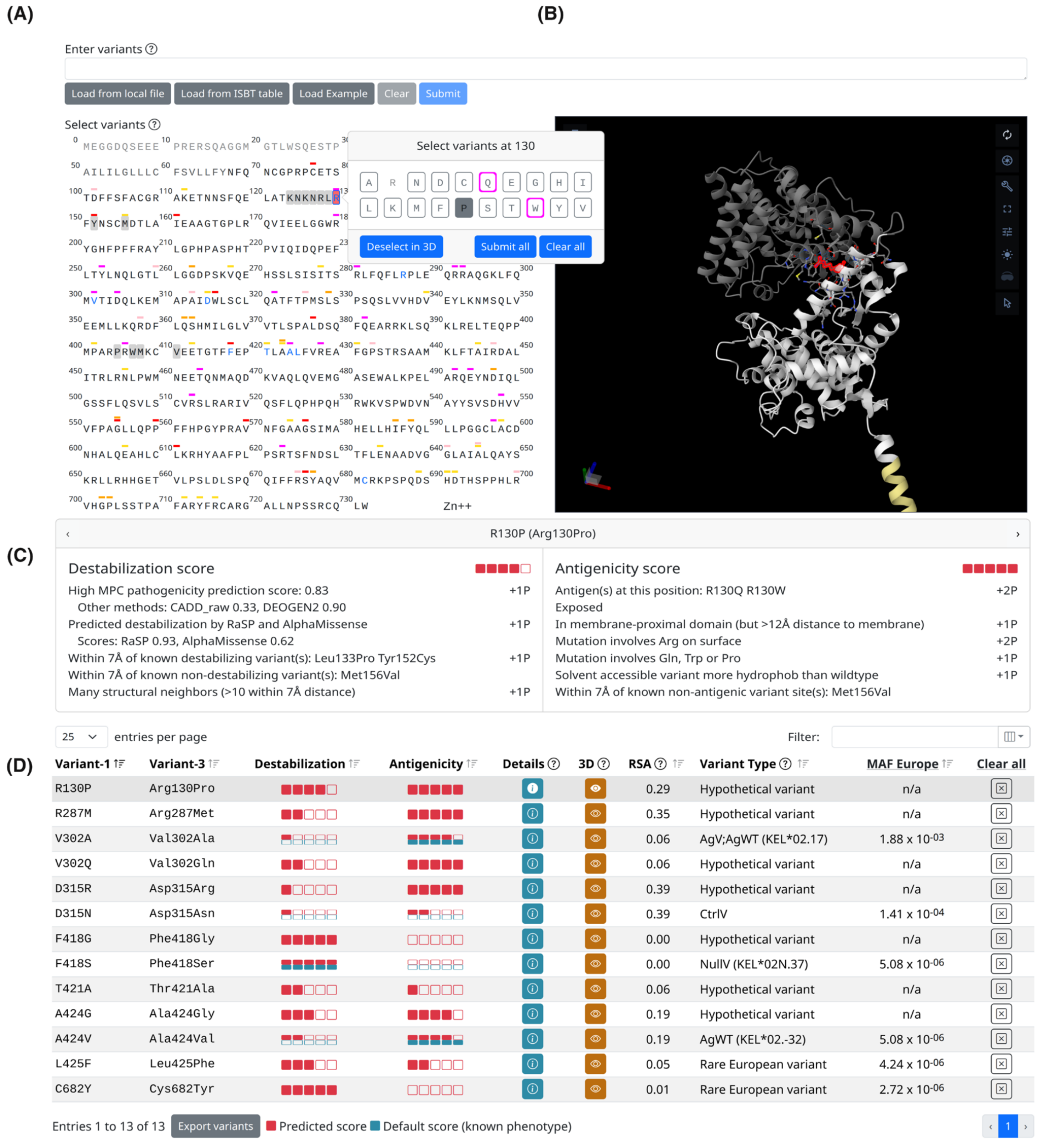
Screenshot from the Kell‐Score web resource. (A) Sequence panel for selecting amino acid positions and their variants, as text‐input, upload, drop‐down menu for International Society of Blood Transfusion (ISBT) alleles or through a pop‐up window. Colour schemes for amino acid positions highlight variant positions with known phenotypes as previously applied.^4^ (B) Kell protein structural model for viewing amino acid positions. The zoom‐in displays the selected and surrounding amino acids, which are also at this point highlighted grey in the sequence. (C) Description of variant features that were selected by the teal info button. (D) The prediction scores listed in red, and in teal the default scores for variants with a known phenotype. The orange icon in the 3D column activates the protein structural visualization of the position. All columns in the table can be sorted and filtered.

Following selection, a table is generated (Figure 1D) that visualizes (i) a prediction score for each phenotype depicted as five red squares (scores in teal colour indicate default scores of known phenotypes); (ii) relative solvent accessibility (RSA); (iii) the variant type, for example, either of an ISBT variant category including allele name^4^ or rare or not yet detected in the European population, or a hypothetical mutation as inferred from the (iv) minor allele frequency (MAF) of the European/non‐Finnish population as derived from the gnomAD database.^8^ By selecting the teal icon in the Details column, a text box opens (Figure 1C) to provide specific information about the variant position and the origin of conditional sub‐scores that sum up to the final score of maximum five. If the variant is given a default score, for example, a variant with a known ISBT phenotype, conditional subscores are indicated as ‘Notional subscores’. An eye‐shaped orange icon in the 3D column activates the protein structural visualization of the position. Every column in the table can be sorted and filtered for prioritizing entries.

The web interface was designed as a single‐page web application and implemented using the Ember.js (https://emberjs.com/) JavaScript web application framework utilizing design templates of the Bootstrap 5 toolkit (https://getbootstrap.com/). The protein visualization is generated by a Mol* plugin instance of the JavaScript library Mol* (https://github.com/molstar/molstar).^7^ Communication between visualization and sequence input is realized using the Mol* plugin's application programming interface (API) and contemporary software design patterns like data binding and modifiers.

Due to the lack of an experimental high‐resolution Kell structure, the method relies on a predicted protein model and is therefore inherently limited in its accuracy regarding 3D structural context and dynamics of individual residues. However, we provide calculated scores also for known Kell antigens and K_0_ phenotype and find that our predictions match well with their phenotype (Figure 1).

To conclude, users of the Kell web resource are provided with a protein sequential and structural viewer that allows them to analyse any single protein mutation in molecular detail and provides an estimate of the molecular consequence of the variant, taking into account the 3D context and all known ISBT phenotypes. In addition, researchers can prioritize variants from any number of entries by ranking destabilization and antigenicity prediction scores.

## AUTHOR CONTRIBUTIONS

Gabriele Mayr designed and performed the research study, conceptualized the web interface, analysed the data and drafted the manuscript. Marcus Schmöhl designed and implemented the webpage. Maike Bublitz analysed the data and drafted the manuscript. Christoph Gassner analysed the data. Andre Franke conceptualized the web interface. All authors read and approved the manuscript.

## FUNDING INFORMATION

This study is an outcome of the Deutsche Forschungsgemeinschaft (DFG) Collaborative Research Center 1182 ‘Origin and Function of Metaorganisms’ (https://www.metaorganism‐research.com, Project number: 261376515, Projects A2.2 and INF) and received infrastructure support from the DFG Excellence Cluster 2167 ‘Precision Medicine in Chronic Inflammation’ (PMI; Project number: 390884018). The Institute of Translational Medicine of the Private University in the Principality of Liechtenstein is financially supported by the Hans Groeber‐Stiftung, Vaduz, Principality of Liechtenstein and the Tarom Foundation, Schaan, Principality of Liechtenstein.

## CONFLICT OF INTEREST STATEMENT

Christoph Gassner acts as a consultant to Inno‐Train GmbH, Kronberg im Taunus, Germany, a provider of genotyping kits for molecular blood group diagnostics since 1998. CG holds the European and US patents P3545102 and US20190316189 on the ‘Determination of the genotype underlying the S‐s‐U‐ phenotype of the MNSs blood group system’. No other conflict of interest is declared by any of the authors.

## Supporting information


Table S1.

Table S2.

## Data Availability

Data are available in the Supporting Information of this article.
